# The cytology of molluscum contagiosum mimicking skin adnexal tumor

**DOI:** 10.4103/0970-9371.70753

**Published:** 2010-04

**Authors:** Jayashree Krishnamurthy, Divya Kota Nagappa

**Affiliations:** Department of Pathology, Medical College (VIMS), Bellary - 583 104, Karnataka, India

**Keywords:** Cytoplasmic inclusions, fine needle aspiration cytology, molluscum contagiosum

## Abstract

Molluscum contagiosum is a cutaneous viral infection presenting as multiple, umbilicated papules and vesicles. The cytology of molluscum contagiosum in an 11-year-old boy, which presented atypically as a solitary nodule over the right cheek, mimicking a skin adnexal tumor is reported here. Fine needle aspiration cytology plays a vital role in establishing the correct diagnosis of clinically unsuspected cases, and hence, the proper management of such lesions. The cytology of molluscum contagiosum is characterized by the presence of numerous large intracytoplasmic basophilic bodies that push the host nucleus to the periphery, giving a signet ring appearance to the superficial epidermal cells.

## Introduction

Molluscum contagiosum is an infectious disease of human skin, characterized by the formation of multiple and discrete cutaneous epithelial nodules averaging about 2 millimetres in diameter.

An unusual case of molluscum contagiosum presenting as a clinically solitary solid nodule diagnosed by fine needle aspiration cytology is reported here.

## Case Report

An 11-year-old boy presented with a solitary nodule over the right cheek which had been there for the last six months. Local examination revealed a 8×6 mm solid, firm, non tender and immobile swelling over the right cheek, below the lower eyelid.

The general physical and systemic examinations were normal. The routine investigations and the blood serology were within normal limits. Patient was immunocompetent. A skin adnexal tumor was suspected clinically.

**Figure 1:**
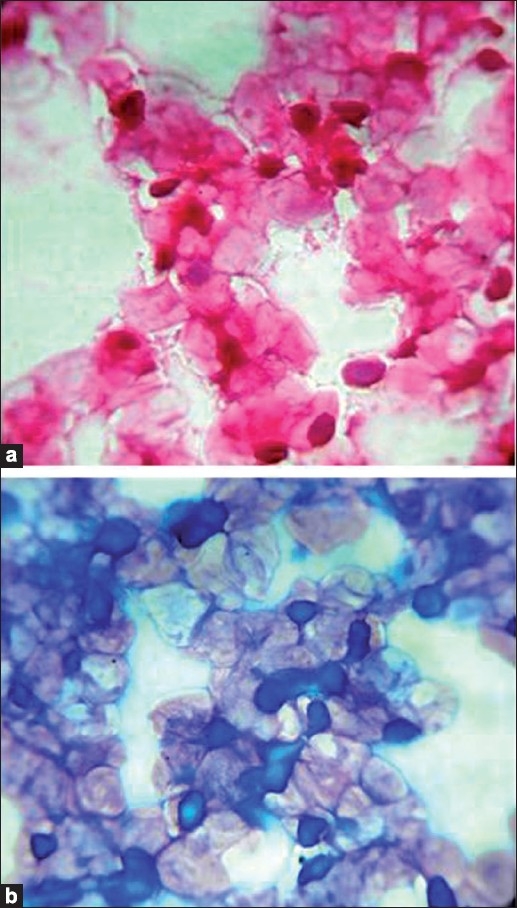
Smears showing intracytoplasmic inclusions pushing the nucleus to periphery (a), (H and E, ×400); (b), (MGG, ×400)

Fine needle aspiration of the right cheek nodule was done using a 24-gauge needle and 10 ml syringe, and it provided a scanty aspirate. The smears were stained with hematoxylin-eosin (H and E) and May-Grünwald-Giemsa (MGG) stains. The cytology of the smears revealed numerous benign squamous cells, with granular intracytoplasmic eosinophilic inclusion bodies, pushing the nucleus to the periphery [Figure 1]. The cytological diagnosis of molluscum contagiosum was suggested and the diagnosis was confirmed by a histopathological examination of the lesion.

## Discussion

Molluscum contagiosum was first described as a clinical entity in 1871 by Bateman.[1] It was studied by various authors-Lipschiitz, Von Prowazek, Kuznitsky, Mac callum.[2]

Molluscum contagiosum is a cutaneous infection produced by molluscum contagiosum virus, specific to humans, present world wide and passed by direct skin-to-skin contact.[3] It affects children, sexually active adults, immunosuppressed persons especially those with human immunodeficiency virus infection, patients with atopic dermatitis, malignancies, those on steroids and other immunosuppressive drugs.[4]

Molluscum contagiosum clinically presents as small, firm, smooth surfaced, dome shaped pearly papules averaging 3-5 mm in diameter, with a central umbilication, distributed over face, trunk and extremities. Atypical presentations like cellulitis, abscesses, molluscum contagiosum with metaplastic ossification and giant molluscum contagiosum are reported.[4–6]

The characteristic cytologic feature of molluscum contagiosum is the presence of molluscum bodies, as described by Handerson and Pattersonin 1891, in the enlarged superficial cells of the epidermis.[2] Molluscum bodies, also called Henderson–Patterson bodies, are large, round cytoplasmic inclusions (within the enlarged cells of epidermis), which push the nucleus to the periphery.[7] Molluscum bodies present as minute, ovoid, eosinophilic structures in the cells of stratum malphigi. At the level of the wide, poorly defined granular layer, the staining reaction of molluscum bodies changes from eosinophic to basophilic. Molluscum bodies are the viral particles which develop about and within the cytoplasmic vacuoles, which are regarded as the cellular response to the presence of living foreign body.[2]

Special stains like phosphotungstic acid-hematoxylin preparation and carbol-anilin-fuchsin after mordanting with potassium permanganate are used to demonstrate molluscum bodies.[2]

Clinical differential diagnoses of molluscum contagiosum include folliculitis, warts, cryptococcosis, nevi and skin adnexal tumor like syringoma, basal cell epithelioma, keratoacanthoma.[4] A cytological examination helps to differentiate these lesions to arrive at a correct diagnosis.

## Conclusion

Molluscum contagiosum presenting as a solitary solid nodule is unusual. In such unsuspected cases, the cytological diagnosis of molluscum contagiosum is suggested by demonstrating the characteristic molluscum bodies in the aspirated material.
